# Vascular Inflammation as a Therapeutic Target in COVID-19 “Long Haulers”: HIITing the Spot?

**DOI:** 10.3389/fcvm.2021.643626

**Published:** 2021-03-19

**Authors:** Regitse Højgaard Christensen, Ronan M. G. Berg

**Affiliations:** ^1^Centre for Physical Activity Research, Rigshospitalet, University of Copenhagen, Copenhagen, Denmark; ^2^Department of Cardiology, Rigshospitalet, University of Copenhagen, Copenhagen, Denmark; ^3^Department of Biomedical Sciences, Faculty of Health and Medical Sciences, University of Copenhagen, Copenhagen, Denmark; ^4^Department of Clinical Physiology, Nuclear Medicine & Positron Emission Tomography (PET), Rigshospitalet, University of Copenhagen, Copenhagen, Denmark; ^5^Neurovascular Research Laboratory, Faculty of Life Sciences and Education, University of South Wales, Newport, United Kingdom

**Keywords:** COVID-19 complications, high-intensity exercise, cardiovascular disease, systemic inflammation, exercise

## Background

In the wake of the first wave of the ongoing global pandemic, it has become imminently clear that coronavirus disease 2019 (COVID-19) has brought with it a whole new clinical syndrome: “long COVID” (1, 2). Hence, after recovery from the acute viral infection, a remarkably large proportion of patients, who initially coined themselves “long haulers” in social media-based patient communities for COVID-19 survivors suffer from persistent and often invalidating symptoms, including dyspnoea, chest pain, tachycardia, post-viral brain fog, exercise intolerance, and extreme fatigue to mention a few (3, 4). According to recent studies ~10% of all individuals infected with the causative acute respiratory syndrome-coronavirus-2 (SARS-CoV-2), and as many as nine out of 10 patients that have required hospitalization because of COVID-19 develop long COVID that persists for at least 4 months, according to the currently available data (4). Time will tell whether the symptoms associated with long COVID are transient or ever-lasting phenomena.

Long COVID will expectedly have a huge impact on the morbidity burden and quality of life in many COVID-19 survivors in the future, and when considering the extent of the global pandemic with currently more than 40 million verified cases, it will expectedly have substantial consequences, both in terms of economic cost and health care capacity throughout the world. It is thus widely recognized that there is an impending need for implementing evidence-based patient-tailored safe and effective rehabilitation schemes, but due to the paucity of data on this, the structure and specificity of such schemes remain obscure. While it is widely recognized that some exercise is better than none and more intense exercise is superior to less intense exercise, opinion papers and guidelines published over the past year have consistently refuted high-intensity interval training (HIIT) as an option for rehabilitation after COVID-19 (5–10). On the basis of the known pathophysiology of COVID-19 and the physiological effects of HIIT, we will however argue in favor of the opposite stand, that is, that HIIT should be considered as one of the rehabilitation interventions of choice for alleviating or even reversing the symptoms of long COVID.

## COVID-19 IS (ALSO) a Vascular Disease

Even though COVID-19 is primarily a viral pneumonia, its multiorgan involvement, both in the acute phase and when considering the persistent systems in long COVID, stresses that this is far from the whole story. Over the past months, several studies have highlighted the presence of a substantial vascular component in the pathophysiology of the disease (11–14). Indeed, COVID-19 is associated with severe vascular inflammation, both in the pulmonary and extrapulmonary vasculature, both on the macro- and microvascular level (11). This involves diffuse endothelial damage with pyroptosis and apoptosis as well as a procoagulant change of the vascular endothelium. Consequently, both pulmonary and extrapulmonary thromboembolism are common complications, that may both determine the initial clinical presentation and the long-term consequences of COVID-19 in many patients (15).

The main mechanisms of the universal vascular component of COVID-19 may both involve the mode of entry of the virus into host cells and the immune response to the virus. The causative severe acute respiratory syndrome coronavirus 2 (SARS-CoV-2) invades host endothelial cells through endocytosis which is facilitated by the angiotensin converting enzyme 2 receptor and the transmembrane protease serine 2 which are expressed in practically all organs throughout the body (16).

In terms of the immune response, a type 3 hypersensitivity reaction has been reported to contribute to vascular inflammation in COVID 19, at least in some cases (17). This type of immune reaction takes place when an excess or slight excess of soluble antigens lead to the accumulation of immune complexes, which then precipitate inside the tissues, in particular blood vessels, where they may cause so-called “leukocytoclastic vasculitis,” which is a procoagulant condition that affects both the macro- and microvasculature.

Another immune mechanism, which is probably important regardless of whether a type 3 reaction takes place, is the highly proinflammatory cytokine response to SARS-CoV-2, which is prominent both in milder and very severe cases, and which some have designated a “cytokine storm” (18, 19). This involves vast elevations in the classical pro-inflammatory cytokines, TNF-α and IL-1β, which have prominent effects on the endothelium. Hence, TNF-α facilitates the development of a procoagulant endothelium by increasing the expression of endothelial cellular adhesion molecules and genes critical for coagulation, such as tissue factor and decreased thrombomodulin, resulting in a pro-thrombotic state (20, 21). Moreover, TNF-α suppresses endothelial nitric oxide synthase and cyclooxygenase 1, which further compounds endothelial dysfunction (22). Furthermore, IL-1β, which is a downstream cytokine of TNF-α in the initial cytokine cascade triggered by an invading pathogen, is a potent trigger of vascular inflammation, among other things by enhancing monocyte and leukocyte infiltration in the vascular wall. This has most convincingly been demonstrated in studies of infants with non-functional IL-1 receptor antagonist (IL-1ra) function and thus uninhibited IL-1β signal transduction, which leads to severe universal vasculitis (23, 24).

In the following sections we will argue that because the multiorgan involvement of COVID-19 may largely reflect universal vascular inflammation, HIIT is an alluring contender for alleviating and perhaps preventing long COVID.

## The Anti-inflammatory Effect of Exercise

**Figure 1:**
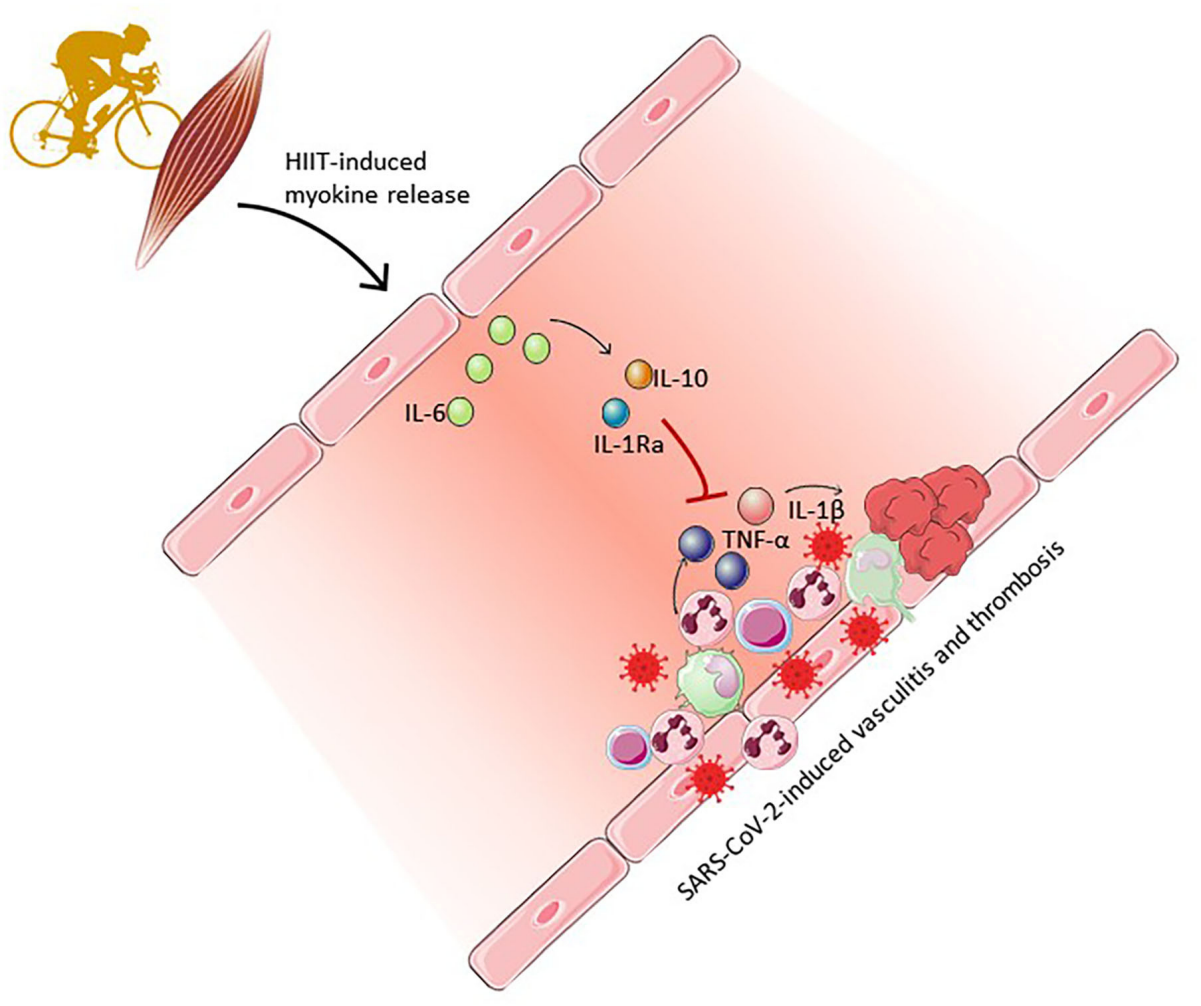
High-intensity interval training (HIIT)-induced myokines (IL-6, IL-10 and IL-1Ra) may counter-act systemic vasculitis through an anti-inflammatory response, namely inhibition of TNFα and IL-1β-mediated activation of pro-coagulant and pro-inflammatory pathways. Clipart provided by Servier Medical Art (60).

Physical exercise is a fundamental physiological stressor that is capable of inducing ubiquitous adaptations in nearly all cells, in nearly all tissues and organs (25). This involves the skeletal muscle “secretome” of myokines that are released from contracting skeletal muscle, and which exerts various functions through autocrine, paracrine, and endocrine functions, including marked immunomodulatory effects (Figure 1) (26). To this end, the low-grade inflammation, which is a common manifestation of aging has been demonstrated to be reversed by exercise of both moderate to strenuous intensity in randomized controlled trials in the elderly (27). Of note, IL-6 is the first detectable myokine released into the bloodstream during exercise. This is triggered by contraction-induced glycogen depletion in skeletal muscle and its concentration in blood increases exponentially depending on the intensity and duration of exercise (25). Therefore, exercise modalities involving large muscle groups produce the greatest IL-6 response. HIIT regimens or marathons can result in IL-6 increase of 100-fold, although increases of 2–10-fold are more common in exercise regimes of more moderate intensity or duration (28).

Once released, recent studies indicate that IL-6 directly stimulates cardiac exercise adaptations (29) and also affects the vasculature by mobilizing natural killer and dendritic cells to the blood stream (30), which are critically involved in viral clearance. The principal immunomodulatory function of IL-6 released during exercise is however to stimulate the release of IL-10 and IL-1ra by monocytes (31), while also reducing the expression of genes encoding several pro-inflammatory cytokines, including TNF-α and IL-1β. IL-10 also directly inhibits the synthesis of TNF-α (32) while IL-1ra inhibits IL-1β signaling. Additionally, IL-10 negatively interferes with tissue factor expression, thus exerting an anti-coagulant effect in the vasculature (Figure 1) (33).

By increasing viral clearance, while also aberrating TNF-α and IL-1β signaling, and alleviating the associated procoagulant state, exercise may thus reduce vascular inflammation in COVID-19.

## HIIT: Is It Effective and/or Safe in COVID-19?

Given that the anti-inflammatory effects of exercise depends critically on the intensity of exercise, intense modalities that involve large muscle groups, such as HIIT protocols, have the potential to produce marked anti-inflammatory effects in target tissues in a time-efficient fashion (28, 34, 35).

HIIT has become increasingly popular in various rehabilitation schemes in patients with lung diseases, mostly because patients with respiratory symptoms are often unable to engage in classical continuous exercise regimens at an intensity sufficient to induce a training adaptation, but during HIIT relatively high intensities are often tolerated (36). Another advantage of HIIT, which is also a benefit in the scientific study of exercise adaptations, is its highly standardized and reproducible nature and that it evokes measurable physiological adaptations much faster than continuous training, i.e., within 2 weeks in healthy volunteers (36). Hence, although an acute HIIT bout elicits apparently similar plasma IL-6 as an iso-energetic continuous exercise bout, the higher intensities and total workloads that may be tolerated during HIIT in various disease states compounds the exercise-induced anti-inflammatory effects (37, 38). Hence, HIIT has been shown to reduce disease-related TNF-α in an animal model of diabetes (39), and furthermore has specific suggested effects related to vascular inflammation, including reduced chemokine chemotaxis and enhanced endothelial repair reported in reviews and meta-analyses conducted on diverse populations of both normal overweight and obese individuals (40–42). This may both reflect the imminent effects of the high-intensity intervals on the IL-6 response as well as on the vasculature *per se*, i.e., due to the pronounced changes in vascular shear stress between intervals (43).

Of all the potential exercise interventions that may be prescribed in COVID-19, HIIT is nonetheless the most controversial. Several aspects of HIIT have been highlighted to disfavor it in this context, including presumed immunosuppressive effects that could increase viral susceptibility and decrease viral clearance (5, 44) and the potential risk of sudden cardiac arrest due to COVID-19-induced residual cardiovascular pathology (45). Due to the latter, the American College of Sports Medicine (ASCM) and experts endorsed by the section of Sports Cardiology & Exercise of the European Association of Preventive Cardiology (EAPC) have recommend that even athletes accustomed to high exercise intensities should resume to exercise only after a complete cardiovascular evaluation and in a gradual manner following a COVID-19 infection (6–10).

Concerns relating to viral susceptibility and clearance are directly contradicted by the known effects of exercise on immune function, including the effects on NK and dendritic cells described above (30). Accordingly, others have also stressed the potential of HIIT as a means to enhance immune surveillance and regulation while also exerting anti- rather than pro-inflammatory effects in COVID-19 survivors (46, 47).

In terms of the concerns of increasing the risk of adverse cardiovascular outcomes by HIIT in COVID-19 survivors, other reports suggest otherwise (48). Hence, a recent, admittedly small retrospective study of 28 discharged COVID-19 survivors reported that rehabilitation triggered by HIIT, with endurance training at the maximum tolerated exercise load was both safe and feasible (49). To this end HIIT has successfully been implemented as a rehabilitation strategy in other “high risk” populations, as demonstrated in larger studies on patients at risk or with prevalent ischaemic heart disease, heart failure, chronic obstructive pulmonary disease, cystic fibrosis, and asthma with effects on parameters such as cardiorespiratory fitness (VO_2_ peak) and exercise capacity with few reports of severe adverse events, even in patients with left ventricular assist devices (36, 41, 50–59). The rate of cardiovascular complications has been reported of 1 per 23,182 h of high-intensity exercise (51) and later studies have confirmed that HIIT is safe in patients with cardiovascular disease (53). As of now, no studies have thus provided any documentation to indicate that high intensity exercise regimes such as HIIT are not safe in COVID-19 survivors.

## Conclusion

While the major focus in handling the burgeoning COVID-19 pandemic has hitherto been on reducing the spread of disease and mortality rates, the startlingly high prevalence and severity of long COVID in survivors heralds an aftermath of similar proportions. This may put health care systems throughout the world on the spot in the years to come, and clinical studies that seek to identify and implement effective rehabilitation strategies are thus of utmost importance. We thus believe that the following questions should be addressed by such studies in the very near near future: “When should HIIT be initiated in COVID-19 patients?,” “Which specific HIIT protocol should be instigated in COVID-19 patients?” and “What are the effects on HIIT-based rehabilitation on cardio-pulmonary function, symptom burden, and quality of life in patients with long COVID?”. HIIT may comprise a valuable component of the rehabilitation intervention in this context, given that its anti-inflammatory effects may target the prominent disease-specific vascular inflammation that is likely a substantial pathogenetic component of the “long haul” of COVID-19.

## Author Contributions

RC and RB conceived and wrote the initial draft of the manuscript. All authors provided critical input at all stages, and were involved in drafting and editing subsequent versions of the manuscript, read, and approved the final version of the manuscript.

## Conflict of Interest

The authors declare that the research was conducted in the absence of any commercial or financial relationships that could be construed as a potential conflict of interest.

## References

[1] Alwan NA Attree E Blair JM Bogaert D Bowen MA Boyle J. From doctors as patients: a manifesto for tackling persisting symptoms of covid-19. BMJ. (2020) 370:m3565. 10.1136/bmj.m3565 32933949

[2] Bos LDJ Brodie D Calfee CS. Severe COVID-19 infections—knowledge gained and remaining questions. JAMA Intern Med. (2020) 181:9–11. 10.1001/jamainternmed.2020.6047 32945833

[3] Rubin R. As their numbers grow, COVID-19 “Long Haulers” stump experts. JAMA. (2020) 1:23–5. 10.1001/jama.2020.17709 32965460

[4] Carfi A Bernabei R Landi F. Persistent symptoms in patients after acute COVID-19. JAMA. (2020) 324:603–5. 10.1001/jama.2020.12603 32644129 PMC7349096

[5] Rahmati-Ahmadabad S. Exercise against SARS-CoV-2 (COVID-19): does workout intensity matter? A mini review of some indirect evidence related to obesity). Obes Med. (2020) 19:100245. 10.1016/j.obmed.2020.100245 32342019 PMC7184978

[6] Bhatia RT Marwaha S Malhotra A Iqbal Z Hughes C Börjesson M. Exercise in the severe acute respiratory syndrome Coronavirus-2 (SARS-CoV-2) era: a question and answer session with the experts endorsed by the section of sports cardiology & exercise of the European association of preventive cardiology (EAPC). Eur J Prev Cardiol. (2020) 27:1242–51. 10.1177/2047487320930596 32475157 PMC7717284

[7] Verwoert GC de Vries ST Bijsterveld N Willems AR vd Borgh R Jongman JK. Return to sports after COVID-19: a position paper from the dutch sports cardiology section of the Netherlands society of cardiology. Netherlands Hear J. (2020) 28:391–5. 10.1007/s12471-020-01469-z 32662058 PMC7357275

[8] Dores H Cardim N. Return to play after COVID-19: a sport cardiologist's view. Br J Sports Med. (2020) 54:8–9. 10.1136/bjsports-2020-102482 32381502

[9] Denay KL Breslow RG Turner MN Nieman DC Roberts WO Best TM. ACSM call to action statement: COVID-19 considerations for sports and physical activity. N Engl J Med. (2020) 383:120–8. 10.1249/JSR.0000000000000739 32769667

[10] Kennedy FM Sharma S. COVID-19, the heart and returning to physical exercise. Occup Med. (2020) 70:467–9. 10.1093/occmed/kqaa154 32816003 PMC7454837

[11] Ackermann M Verleden SE Kuehnel M Haverich A Welte T Laenger F. Pulmonary vascular endothelialitis, thrombosis, and angiogenesis in Covid-19. N Engl J Med. (2020) 383:120–8. 10.1056/NEJMoa2015432 32437596 PMC7412750

[12] Iba T Connors JM Levy JH. The coagulopathy, endotheliopathy, and vasculitis of COVID-19. Inflamm Res. (2020) 69:1181–9. 10.1007/s00011-020-01401-6 32918567 PMC7486586

[13] Vacchi C Meschiari M Milic J Marietta M Tonelli R Alfano G. COVID-19-associated vasculitis and thrombotic complications: from pathological findings to multidisciplinary discussion. Rheumatology. (2020) 59:e147–50. 10.1093/rheumatology/keaa581 32968761 PMC7543638

[14] Verdoni L Mazza A Gervasoni A Martelli L Ruggeri M Ciuffreda M. An outbreak of severe kawasaki-like disease at the italian epicentre of the SARS-CoV-2 epidemic: an observational cohort study. Lancet. (2020) 395:1771–8. 10.1016/S0140-6736(20)31103-X 32410760 PMC7220177

[15] Madjid M Safavi-Naeini P Solomon SD Vardeny O. Potential effects of Coronaviruses on the cardiovascular system: a review. JAMA Cardiol. (2020) 10:1–10. 10.1001/jamacardio.2020.1286 32219363

[16] Libby P. The heart in COVID-19: primary target or secondary bystander? JACC Basic Transl Sci. (2020) 5:537–42. 10.1016/j.jacbts.2020.04.001 32292847 PMC7151324

[17] Roncati L Ligabue G Fabbiani L Malagoli C Gallo G Lusenti B. Type 3 hypersensitivity in COVID-19 vasculitis. Clin Immunol. (2020) 217:108487. 10.1016/j.clim.2020.108487 32479986 PMC7256503

[18] Ronit A Berg RMG Bay J Haugaard AK Ahlström MG Burgdorf KS. Compartmental immunophenotyping and cytomorphology in COVID-19 ARDS: a case series. J Allergy Clin Immunol. (2020) 147:81–91. 10.1016/j.jaci.2020.09.009 32979342 PMC7581505

[19] Cao X. COVID-19: immunopathology and its implications for therapy. Nat Rev Immunol. (2020) 20:269–70. 10.1038/s41577-020-0308-3 32273594 PMC7143200

[20] Tremoli E Camera M Toschi V Colli S. Tissue factor in atherosclerosis. Atherosclerosis. (1999) 144:273–83. 10.1016/S0021-9150(99)00063-5 10407489

[21] Hot A Lenief V Miossec P. Combination of IL-17 and TNFα induces a pro-inflammatory, pro-coagulant and pro-thrombotic phenotype in human endothelial cells. Ann Rheum Dis. (2012) 71:768–76. 10.1136/annrheumdis-2011-200468 22258491

[22] Vallance P Collier J Bhagat K. Infection, inflammation, and infarction: does acute endothelial dysfunction provide a link? Lancet. (1997) 349:1391–2. 10.1016/S0140-6736(96)09424-X 9149715

[23] Dinarello CA Simon A van der Meer JWM. Treating inflammation by blocking interleukin-1 in a broad spectrum of diseases. Nat Rev Drug Discov. (2012) 11:633–52. 10.1038/nrd3800 22850787 PMC3644509

[24] Dinarello CA. Interleukin-1 in the pathogenesis and treatment of inflammatory diseases. Blood. (2011) 117:3720–32. 10.1182/blood-2010-07-273417 21304099 PMC3083294

[25] Pedersen BK Febbraio MA. Muscle as an endocrine organ: focus on muscle-derived interleukin-6. Physiol Rev. (2008) 88:1379–406. 10.1152/physrev.90100.2007 18923185

[26] Pedersen BK. Muscle as a secretory organ. Compr Physiol. (2013) 3:1337–62. 10.1002/cphy.c120033 23897689

[27] Woods JA Wilund KR Martin SA Kistler BM. Exercise, inflammation and aging. Aging Dis. (2012) 3:130–40. 22500274 PMC3320801

[28] Fischer C. Interleukin-6 in acute exercise and training: what is the biological relevance. Exerc Immunol Rev. (2006) 12:6–33. 17201070

[29] Christensen RH Wedell-Neergaard AS Lehrskov LL Legaard GE Dorph EB Larsen MK. Effect of aerobic and resistance exercise on cardiac adipose tissues: secondary analyses from a randomized controlled trial. JAMA Cardiol. (2019) 4:778–87. 10.1001/jamacardio.2019.2074 31268469 PMC6613292

[30] Bay ML Heywood S Wedell-Neergaard A Schauer T Lehrskov LL Christensen RH. Human immune cell mobilization during exercise – effect of IL-6 receptor blockade. Exp Physiol. (2020) 105:2086–98. 10.1113/EP088864 33006190

[31] Ostrowski K Rohde T Asp S Schjerling P Pedersen BK. Pro- and anti-inflammatory cytokine balance in strenuous exercise in humans. J Physiol. (1999) 515(Pt 1):287–91. 10.1111/j.1469-7793.1999.287ad.x 9925898 PMC2269132

[32] Starkie R Ostrowski SR Jauffred S Febbraio M Pedersen BK. Exercise and IL-6 infusion inhibit endotoxin-induced TNF-alpha production in humans. FASEB J. (2003) 17:884–6. 10.1096/fj.02-0670fje 12626436

[33] Pedersen BK. Anti-inflammatory effects of exercise: role in diabetes and cardiovascular disease. Eur J Clin Invest. (2017) 47:600–11. 10.1111/eci.12781 28722106

[34] Helge JW Stallknecht B Pedersen BK Galbo H Kiens B Richter EA. The effect of graded exercise on IL-6 release and glucose uptake in human skeletal muscle. J Physiol. (2003) 546:299–305. 10.1113/jphysiol.2002.030437 12509497 PMC2342463

[35] Cullen T Thomas AW Webb R Hughes MG. Interleukin-6 and associated cytokine responses to an acute bout of high-intensity interval exercise: the effect of exercise intensity and volume. Appl Physiol Nutr Metab. (2016) 41:803–8. 10.1139/apnm-2015-0640 27377137

[36] Sawyer A Cavalheri V Hill K. Effects of high intensity interval training on exercise capacity in people with chronic pulmonary conditions: a narrative review. BMC Sports Sci Med. (2020) 12:22. 10.1186/s13102-020-00167-y 32257221 PMC7106755

[37] de Souza DC Matos VAF dos Santos VOA Medeiros IF Marinho CSR Nascimento PRP. Effects of high-intensity interval and moderate-intensity continuous exercise on inflammatory, leptin, IgA, and lipid peroxidation responses in obese males. Front Physiol. (2018) 9:1–9. 10.3389/fphys.2018.00567 29875681 PMC5974092

[38] Peake JM Tan SJ Markworth JF Broadbent JA Skinner TL Cameron-Smith D. Metabolic and hormonal responses to isoenergetic high-intensity interval exercise and continuous moderate-intensity exercise. Am J Physiol Endocrinol Metab. (2014) 307:E539–52. 10.1152/ajpendo.00276.2014 25096178

[39] Kim JS Lee YH Kim JC Ko YH Yoon CS Yi HK. Effect of exercise training of different intensities on anti-inflammatory reaction in streptozotocin-induced diabetic rats. Biol Sport. (2014) 31:73–9. 10.5604/20831862.1093775 25187675 PMC3994589

[40] Li Y Liu D Wu H. HIIT: a potential rehabilitation treatment in COVID-19 pneumonia with heart disease. Int J Cardiol. (2020) 320:186. 10.1016/j.ijcard.2020.07.030 32721412 PMC7833490

[41] Batacan RB Duncan MJ Dalbo VJ Tucker PS Fenning AS. Effects of high-intensity interval training on cardiometabolic health: a systematic review and meta-analysis of intervention studies. Br J Sports Med. (2017) 51:494–503. 10.1136/bjsports-2015-095841 27797726

[42] Pal S Radavelli-Bagatini S Ho S. Potential benefits of exercise on blood pressure and vascular function. J Am Soc Hypertens. (2013) 7:494–506. 10.1016/j.jash.2013.07.004 23992766

[43] Williams JS Del Giudice M Gurd BJ Pyke KE. Reproducible improvement in endothelial function following two separate periods of high-intensity interval training in young men. J Appl Physiol. (2020) 129:725–31. 10.1152/japplphysiol.00054.2020 32790591 PMC7654699

[44] Leandro CG Ferreira E Silva WT Lima-Silva AE. Covid-19 and exercise-induced immunomodulation. Neuroimmunomodulation. (2020) 27:75–8. 10.1159/000508951 32506067 PMC7316658

[45] Baggish AL Levine BD. Icarus and sports after COVID 19: too close to the sun? Circulation. (2020) 142:615–7. 10.1161/CIRCULATIONAHA.120.048335 32516032 PMC7424894

[46] Wang M Baker JS Quan W Shen S Fekete G Gu Y. A preventive role of exercise across the Coronavirus 2 (SARS-CoV-2) pandemic. Front Physiol. (2020) 11:1–8. 10.3389/fphys.2020.572718 33013486 PMC7506115

[47] da Silveira MP da Silva Fagundes KK Bizuti MR Starck É Rossi RC de Resende e Silva DT. Physical exercise as a tool to help the immune system against COVID-19: an integrative review of the current literature. Clin Exp Med. (2020) 21:15–28. 10.1007/s10238-020-00650-3 32728975 PMC7387807

[48] Batatinha HAP Krüger K Neto JCR. Thromboinflammation and COVID-19 : the role of exercise in the prevention and treatment. Front Cardiovasc Med. (2020) 7:8–11. 10.3389/fcvm.2020.582824 33392268 PMC7775570

[49] Hermann M Pekacka-Egli A-M Witassek F Baumgaertner R Schoendorf S Spielmanns M. Feasibility and efficacy of cardiopulmonary rehabilitation after COVID-19. Am J Phys Med Rehabil. (2020) 99:865–9. 10.1097/PHM.0000000000001549 32732746 PMC7406212

[50] Ellingsen Ø Halle M Conraads V Støylen A Dalen H Delagardelle C. High-intensity interval training in patients with heart failure with reduced ejection fraction. Circulation. (2017) 135:839–49. 10.1161/CIRCULATIONAHA.116.022924 28082387 PMC5325251

[51] Rognmo O Moholdt T Bakken H Hole T Mølstad P Myhr NE. Cardiovascular risk of high-versus moderate-intensity aerobic exercise in coronary heart disease patients. Circulation. (2012) 126:1436–40. 10.1161/CIRCULATIONAHA.112.123117 22879367

[52] Keech A Way K Holgate K Fildes J Indraratna P Yu J. HIIT for post-COVID patients within cardiac rehabilitation: response to letter to the editor. Int J Cardiol. (2020) 322:291–2. 10.1016/j.ijcard.2020.08.086 32882289 PMC7456951

[53] Wewege MA Ahn D Yu J Liou K Keech A. High-intensity interval training for patients with cardiovascular disease-is it safe? A systematic review. J Am Heart Assoc. (2018) 7:1–19. 10.1161/JAHA.118.009305 30376749 PMC6404189

[54] Alvarez Villela M Chinnadurai T Salkey K Furlani A Yanamandala M Vukelic S. Feasibility of high-intensity interval training in patients with left ventricular assist devices: a pilot study. ESC Hear Fail. (2020) 8:498–507. 10.1002/ehf2.13106 33205573 PMC7835573

[55] Angadi SS Mookadam F Lee CD Tucker WJ Haykowsky MJ Gaesser GA. High-intensity interval training vs. moderate-intensity continuous exercise training in heart failure with preserved ejection fraction: a pilot study. J Appl Physiol. (2015) 119:753–8. 10.1152/japplphysiol.00518.2014 25190739

[56] Guadalupe-Grau A Aznar-Laín S Mañas A Castellanos J Alcázar J Ara I. Short- and long-term effects of concurrent strength and HIIT training in octogenarians with COPD. J Aging Phys Act. (2017) 25:105–15. 10.1123/japa.2015-0307 27402660

[57] Trachsel LD David LP Gayda M Henri C Hayami D Thorin-Trescases N. The impact of high-intensity interval training on ventricular remodeling in patients with a recent acute myocardial infarction—A randomized training intervention pilot study. Clin Cardiol. (2019) 42:1222–31. 10.1002/clc.23277 31599994 PMC6906981

[58] Gomes Neto M Durães AR Conceição LSR Saquetto MB Ellingsen Ø Carvalho VO. High intensity interval training versus moderate intensity continuous training on exercise capacity and quality of life in patients with heart failure with reduced ejection fraction: a systematic review and meta-analysis. Int J Cardiol. (2018) 261:134–41. 10.1016/j.ijcard.2018.02.076 29572084

[59] Villelabeitia-Jaureguizar K Vicente-Campos D Senen AB Jiménez VH Garrido-Lestache MEB Chicharro JL. Effects of high-intensity interval versus continuous exercise training on post-exercise heart rate recovery in coronary heart-disease patients. Int J Cardiol. (2017) 244:17–23. 10.1016/j.ijcard.2017.06.067 28648356

[60] Les Laboratories Servier. Servier Medical Art. Available online at: http://servier.com/Powerpoint-image-bank (accessed October 21, 2020).

